# Neurological Monitoring and Management for Adult Extracorporeal Membrane Oxygenation Patients: Extracorporeal Life Support Organization Consensus Guidelines

**DOI:** 10.1097/MAT.0000000000002312

**Published:** 2024-11-26

**Authors:** Sung-Min Cho, Jaeho Hwang, Giovanni Chiarini, Marwa Amer, Marta Velia Antonini, Nicholas Barrett, Jan Belohlavek, Jason E. Blatt, Daniel Brodie, Heidi J. Dalton, Rodrigo Diaz, Alyaa Elhazmi, Pouya Tahsili-Fahadan, Jonathon Fanning, John Fraser, Aparna Hoskote, Jae-Seung Jung, Christopher Lotz, Graeme MacLaren, Giles Peek, Angelo Polito, Jan Pudil, Lakshmi Raman, Kollengode Ramanathan, Dinis Dos Reis Miranda, Daniel Rob, Leonardo Salazar Rojas, Fabio Silvio Taccone, Glenn Whitman, Akram M. Zaaqoq, Roberto Lorusso

**Affiliations:** 1 Divisions of Neuroscience Critical Care and Cardiac Surgery Departments of Neurology, Neurosurgery, and Anaesthesiology and Critical Care Medicine, The Johns Hopkins University School of Medicine, 600 N. Wolfe Street, Phipps 455, 21287, Baltimore, MD, USA.; 2 Division of Cardiac Surgery, Department of Surgery, The Johns Hopkins University School of Medicine, Baltimore, MD, USA.; 3 Cardiothoracic Surgery Department, Heart and Vascular Centre, Maastricht University Medical Centre, Cardiovascular Research Institute Maastricht, Maastricht, The Netherlands.; 4 Division of Anaesthesiology, Intensive Care and Emergency Medicine, Spedali Civili University, Affiliated Hospital of Brescia, Brescia, Italy.; 5 Medical/Critical Pharmacy Division, King Faisal Specialist Hospital and Research Center, 11564, Al Mathar Ash Shamali, Riyadh, Saudi Arabia.; 6 Alfaisal University College of Medicine, Riyadh, Saudi Arabia.; 7 Bufalini Hospital, AUSL della Romagna, Cesena, Italy.; 8 Department of Critical Care Medicine, Guy’s and St Thomas’ National Health Service Foundation Trust, London, UK.; 9 2nd Department of Medicine, Cardiology and Angiologiy, General University Hospital and 1st School of Medicine, Charles University, Prague, Czech Republic.; 10 Department of Neurosurgery, University of Florida, Gainesville, Florida, USA.; 11 Division of Pulmonary, and Critical Care Medicine, Department of Medicine, The Johns Hopkins University School of Medicine, Baltimore, MD, USA.; 12 Departments of Surgery and Pediatrics, Creighton University, Omaha, NE, USA.; 13 Programa de Oxigenación Por Membrana Extracorpórea, Hospital San Juan de Dios Santiago, Santiago, Chile.; 14 Medical Critical Care Service, Department of Medicine, Inova Fairfax Medical Campus, Falls Church, VA, USA.; 15 Critical Care Research Group, Adult Intensive Care Services, The Prince Charles Hospital and University of Queensland, Rode Rd, 4032, Chermside, QLD, Australia.; 16 Cardiorespiratory and Critical Care Division, Great Ormond Street Hospital for, Children National Health Service Foundation Trust, London, UK.; 17 Department of Thoracic and Cardiovascular Surgery, Korea University Medicine, Seoul, Republic of Korea.; 18 Department of Anaesthesiology, Intensive Care, Emergency and Pain Medicine, University Hospital Würzburg, Würzburg, Germany.; 19 Cardiothoracic Intensive Care Unit, Department of Cardiac, Thoracic and Vascular Surgery, National University Health System, Singapore, Singapore.; 20 Congenital Heart Center, Departments of Surgery and Pediatrics, University of Florida, Gainesville, FL, USA.; 21 Pediatric Intensive Care Unit, Department of Woman, Child, and Adolescent Medicine, Geneva University Hospital, Geneva, Switzerland.; 22 Department of Pediatrics, Section Critical Care Medicine, Children’s Medical Center at Dallas, The University of Texas Southwestern Medical Center at Dallas, Dallas, TX, USA.; 23 Department of Intensive Care, Erasmus University Medical Center, Rotterdam, The Netherlands.; 24 ECMO Department, Fundacion Cardiovascular de Colombia, Floridablanca, Santander, Colombia.; 25 Department of Intensive Care, Hôpital Universitaire de Bruxelles (HUB), Université Libre de Bruxelles (ULB), Brussels, Belgium.; 26 Department of Anesthesiology, Division of Critical Care, University of Virginia, Charlottesville, VA, USA.

**Keywords:** ECMO, Guidelines, Neuromonitoring, Neurological care, ICU care, Acute brain injury, Stroke, Neurological outcomes

## Abstract

**Background:**

Critical care of patients on extracorporeal membrane oxygenation (ECMO) with acute brain injury (ABI) is notable for a lack of high-quality clinical evidence. Here, we offer guidelines for neurological care (neurological monitoring and management) of adults during and after ECMO support.

**Methods:**

These guidelines are based on clinical practice consensus recommendations and scientific statements. We convened an international multidisciplinary consensus panel including 30 clinician-scientists with expertise in ECMO from all chapters of the Extracorporeal Life Support Organization (ELSO). We used a modified Delphi process with three rounds of voting and asked panelists to assess the recommendation levels.

**Results:**

We identified five key clinical areas needing guidance: (1) neurological monitoring, (2) post-cannulation early physiological targets and ABI, (3) neurological therapy including medical and surgical intervention, (4) neurological prognostication, and (5) neurological follow-up and outcomes. The consensus produced 30 statements and recommendations regarding key clinical areas. We identified several knowledge gaps to shape future research efforts.

**Conclusions:**

The impact of ABI on morbidity and mortality in ECMO patients is significant. Particularly, early detection and timely intervention are crucial for improving outcomes. These consensus recommendations and scientific statements serve to guide the neurological monitoring and prevention of ABI, and management strategy of ECMO-associated ABI.

## Introduction

Extracorporeal membrane oxygenation (ECMO) is increasingly utilized, yet patients receiving ECMO support commonly experience major complications, including acute brain injury (ABI). ABI increases in-hospital mortality by a factor of 2–3.^1,2^ ABI is more common in venoarterial (VA) ECMO than venovenous (VV) ECMO, especially for those with extracorporeal cardiopulmonary resuscitation (ECPR) with 27–32% of ABI during ECMO support (Table 1) despite its survival benefit.^3,4^ Although a protocolized neurological monitoring is shown to improve the detection of ABI, this is limited to a few ECMO centers.^5^ The management of ECMO patients in the intensive care unit (ICU) is not standardized, and neurological monitoring and care vary significantly across ECMO centers, thus, the ICU management of patients with ABI during ECMO lacks high-quality evidence and recommendations.

**Table 1.** Different ABI types and their weighted prevalence during ECMO support (meta-analysis)^6^

Types	Frequency	ECMO configuration
All ABI types	16% (95% CI 13–19%)	VA ECMO (19%); VV ECMO (10%)
Hypoxic-ischemic brain injury	8% (95% CI 3–14%)	VA ECMO (13%); VV ECMO (1%)
Ischemic stroke	7% (95% CI 4–9%)	VA ECMO (10%); VV ECMO (1%)
Intracerebral hemorrhage	7% (95% CI 5–9%)	VA ECMO ECMO (6%); VV ECMO (8%)
Subarachnoid hemorrhage	4% (95% CI 1–9%)	VA ECMO ECMO (11%)*; VV ECMO ECMO (4%)

ABI = acute brain injury, ECMO = extracorporeal membrane oxygenation, VA = venoarterial, VV = venovenous.

* Reported in only one cohort study.

As clinical experience accumulates and ECMO becomes more widely used, clinical guidelines and focused research on neurological monitoring and management of ABI are imperative to enhance ECMO patient care and improve early as well as long-term outcomes. This heterogeneity presents an opportunity to standardize and facilitate neurological care in ECMO.^5^

To establish clinical guidelines on this topic, an international multidisciplinary panel of experts specialized in neurology, critical care, surgery, and other ECMO-related fields was assembled to provide clinical practice consensus recommendations and scientific statements in neurological monitoring and management of adult ECMO patients. These recommendations and statements have been promoted and endorsed by the Extracorporeal Life Support Organization (ELSO). We identified five key clinical areas needing recommendations: (1) neurological monitoring, (2) post-cannulation early physiological targets and their associations with ABI, (3) neurological therapy including medical and surgical intervention, (4) neurological prognostication, and (5) neurological follow-up and outcomes. Here, we present consensus recommendations based on the available evidence and related knowledge gaps warranting further investigations were also identified and summarized (Table 2).

**Table 2.** Key gaps in knowledge and future direction for neurological care of ECMO

*Prevention and early recognition of acute brain injury in ECMO patients*
Universally agreed-upon protocol for the neuromonitoring of ECMO patients
Optimum analgo-sedative medication uses and pharmacokinetics when the patient is on ECMO
The impact of hyperoxemia (PaO_2_ > 300 mmHg) on the development of acute brain injury in ECMO patients
The change in PaCO_2_, specifically in VA ECMO patients, and the development of acute brain injury
The utilization of hypothermia and the optimal temperature target on postcardiac arrest patients supported by ECPR
The blood pressure goal to maintain cerebral perfusion/autoregulation to prevent acute brain injury
*Interventional neurology and neurosurgery*
Standardized approach for managing neurological complications for patients supported by ECMO
Safety of using tPA in patients on ECMO The optimum anticoagulation goal to prevent acute brain injury on ECMO
The safe duration of holding anticoagulation for patients with intracranial hemorrhage and support by VA ECMO
*Neurological prognostication*
The decision on the duration of ECMO support when there is a lack of neurological recovery
*Long-term outcomes and quality of life*
The natural progression of acute brain injury in patients supported by ECMO
The utility of brain imaging findings on patient management and subsequent outcomes
The long-term cognitive and psychological outcomes for patients supported by ECMO
The long-term impact on the society and the caregivers Quality of life for patients with acute brain injury supported by ECMO

ECMO: extracorporeal membrane oxygenation, PaCO_2_: partial pressure of carbon dioxide; PaO_2_: partial pressure of oxygen; tPA: tissue plasminogen activator; VA: venoarterial.

## Methods

### Consensus guideline members

ELSO, an international non-profit consortium of healthcare institutions, researchers, and industry partners, developed this consensus statement. ELSO consists of 611 ECMO centers, with chapters in Europe, Asia–Pacific, North America, Latin America, Southwest Asia, and Africa.

An international multidisciplinary consensus panel of 30 experts, including neurologists, intensivists, surgeons, perfusionists, and other professionals in intensive care medicine with expertise or involvement in ECMO, from all ELSO chapters was assembled.

Each of the five-panel subgroups addressed a pre-selected clinical practice domain relevant to patients admitted to the ICU with ABI (ischemic stroke, ICH, or hypoxic-ischemic brain injury). Invited experts contributed to the guidelines through a three-phase process: (1) a literature search/review of neurological monitoring, management, and neurological ECMO outcomes, (2) summarizing the literature search/review, and (3) developing consensus guidelines using a modified Delphi method. The literature search and review performed comprehensively in PubMed on August 29, 2023 yielded up-to-date evidence on neurological monitoring and management strategies. Five key neurological areas needing recommendations were identified (see Introduction).

### Guideline development

The selected articles were distributed to each subgroup. The subgroups summarized the findings and developed guidelines and recommendations for each subsection. Each subgroup nominated two leaders for cross-subgroup coordination. The consensus guideline members met regularly throughout the year in subgroup and whole-group settings to discuss their progress and reach a consensus on the finalized document. A modified Delphi process with three rounds of voting to assess the recommendation statements was implemented. Strong recommendation, weak recommendation, or no recommendation was defined when > 85%, 75–85%, and < 75% of panelists, respectively, agreed with a recommendation statement. Three rounds of voting and the authors’ comments about the expert consensus guideline appear in Supplemental Tables 1–3. The guidelines and recommendations were summarized and presented as 5 sections: (1) neurological assessment and monitoring; (2) bedside management; (3) interventional neurology, neurosurgery, and neurocritical care; (4) neurological prognostication; and (5) long-term outcome and quality of life.

**Table 3.** Neurological monitoring tools in patients with ECMO support

Neuromonitoring tool	Evidence
Near infrared spectroscopy (NIRS)	Several studies have shown that a large drop in rSO_2_ below baseline has been associated with brain injury.^15,16^ In a systematic review and meta-analysis on the role of rSO_2_ in ECPR, higher pre-cannulation rSO_2_ was associated with reduced mortality and better neurological outcomes.^17^ Asymmetric desaturation (right-left) and the duration of desaturation may be better markers for ABI detection.^15^ A few studies have utilized NIRS to identify disturbances in autoregulation^18,19^
Transcranial doppler ultrasound	Cerebral blood flow velocities and pulsatility index changes may be an early warning of ABI. In a prospective study of 135 patients, the absence of a pulsatility index was associated with a higher frequency of intraparenchymal hemorrhage and a composite bleeding event.^20^ However, caution should be exercised in interpreting the pulsatility index, as in another case series of adult VA ECMO, patients’ low or absent pulsatility index was related to their cardiac output.^21^ Similarly, microembolic signals in transcranial Doppler ultrasound needs further studies, as current evidence lacks correlation with the burden of microembolic signals to ABI^22^
Pupillometry	Automated pupillometry can objectively evaluate pupil size and reactivity and provide reliable prognostication information.^23^ Analysis of neurologic pupil index in 100 ECMO patients showed that a neurologic pupil index < 3 at any time 24–72 h after cannulation has 100% specificity for 90-day mortality, with 0% false positives;^24^ however, no pupillometry data on neurological outcomes exist in ECMO. High opiate doses may affect the reliability of pupillometry assessment
Electroencephalography (EEG)	The American Clinical Neurophysiology Society consensus statement (2015) recommended EEG monitoring for patients on ECMO who are at high risk for neurological complications such as seizures. Early EEG on ECMO can help in identifying non-convulsive seizures with an opportunity for early intervention.^25,26^ Poor background activity on continuous EEG has been associated with poor outcomes on ECMO^27–29^ particularly in high-risk groups such as ECPR^30,31^
Somatosensory evoked potential (SSEP)	SSEP measure of cortical activity in response to stimuli in the peripheral nervous system has been utilized for neurological prognostication in comatose patients (especially for cardiac arrest). Application in the setting of ECMO, particularly in ECPR patients, may have a critical role. In a case series of 13 patients, Cho et al. showed that patients with a delayed response had poor neurological outcomes. However, in a subsequent cohort, the N20 responses remained intact despite poor neurological outcomes.^29^ Further nvestigations are needed to determine the actual value of this diagnostic examination
Head computed tomography (CT)	Most ECMO centers perform head CTs upon new clinical neurological concerns. However, studies of VV ECMO, VA ECMO, and ECPR have reported routine head CT within 24 h post-cannulation to be beneficial for early diagnosis of subclinical intracranial complications, such as large infarctions, hemorrhage, or edema, which may influence decisions on ECMO support management^32–36^. Limitations of head CT include poor sensitivity for detecting acute or small infarctions, and inability to transport hemodynamically unstable patients to the CT scanner. Early cessation and judicious resumption of anticoagulation appeared feasible in the cohort of patients with ECMO-associated ischemic stroke and ICH with the use of serial neuroimaging studies if feasible^37^
Brain magnetic resonance imaging (MRI)	More than 30% of patients do not receive head CT scans during ECMO support due to transport restrictions and the lack of available transport personnel.^7^ The gold standard for diagnosing ABI is represented by 1.5-3 T MRI, which is incompatible with extracorporeal life support circuits and equipment due to safety concerns (heating, migration, and malfunction). Recent advances in low-field (64 mT) and portable MRI technology enable the acquisition of clinically meaningful imaging in the presence of ferromagnetic materials. The safety and feasibility of portable MRI in adults with ECMO support were demonstrated^38^
Serum biomarker	The most studied biomarkers include serum neuron-specific enolase and S100B, with higher values being significant predictors of mortality or neurological complications, especially if measured serially during ECMO support.^39,40^ However, the optimal timing and frequency of measurement, specific threshold values for outcome prediction, and methods to control for confounding effects have not been standardized

ABI: acute brain injury; CT: computed tomography; ECMO: extracorporeal membrane oxygenation: ECPR: extracorporeal cardiopulmonary resuscitation; EEG: electroencephalography; ICH: intracranial hemorrhage; MRI: magnetic resonance imaging; NIRS: Near Infrared Spectroscopy; rSO_2_: regional tissue oxygen saturation; SSEP: somatosensory evoked potential; VA: venoarterial; VV: venovenous.

## Neurological Assessment and Monitoring

### Neurological examination

Serial bedside examination remains the mainstay of neurological assessment in ECMO patients. However, neurological evaluation, especially early after ECMO cannulation, is frequently confounded by sedatives and paralytics, necessitating noninvasive multimodal neurological monitoring in patients with impaired consciousness. The data on ABI timing to ECMO cannulation/support are limited. Therefore, a baseline neurological assessment is recommended before and immediately after cannulation, followed by serial evaluations throughout ECMO support and after weaning. The ideal frequency of neurological examination is not yet established. Daily assessment by a neurologist/neurointensivist (if available) can improve neurological care.^5,7^ More frequent bedside nursing assessment, every 1–4 h based on ABI risk, is reasonable. Particularly, assessing signs of life (such as gasping, pupillary light response, and increased consciousness) is integral to the clinical examination, as these signs observed before, during resuscitation, and while on ECMO support may be associated with improved neurological outcomes.^8^ Historically, the absence of brainstem reflexes with fixed, dilated pupils before cannulation was equated to irreversible ABI and a contraindication to ECMO. However, during cardiopulmonary resuscitation (CPR), fixed and dilated pupils are frequently seen after epinephrine administration, and patients have achieved favorable outcomes despite these findings.^9^

Serial neurological examination should include mental status assessment, brainstem reflexes (pupillary light response and oculocephalic, corneal, and cough/gag reflexes), and motor exam. Standardized scoring tools such as the Glasgow Coma Scale and the Confusion Assessment Method should be used. Assessing the motor response of extremities in neurological examinations is only helpful when analgo-sedation and paralytic is lightened or off. Therefore, neurological exam for spinal cord injury, a rare but devastating injury, is very challenging.^10^ Sensory exams are mostly limited in ECMO patients.

### Sedation

Adequate analgo-sedation is essential to ECMO support and minimizes adverse events.^11^ ECMO circuitry and common concomitant impaired liver or kidney function alter medication pharmacokinetics. Standardized sedation protocols with validated scoring systems, such as the Richmond Agitation Sedation Scale, are recommended. Overall, intermittent (as-needed) analgo-sedation is preferred over continuous infusion. Short-acting, non-benzodiazepine sedatives could be considered.^11^ Daily reassessment of sedation goals, stepwise sedation weaning, and sedation interruptions can improve neurological exams and ABI diagnosis.^11^

### Neurological monitoring

**Figure 1.**
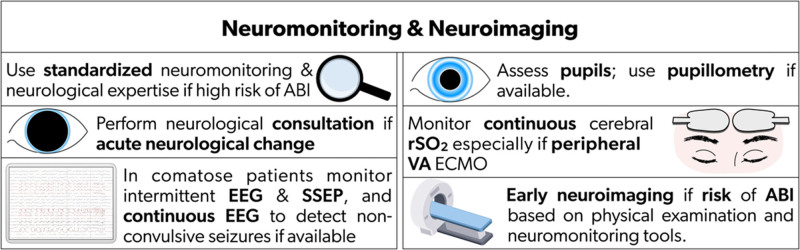
Recommendations for neurological monitoring and neuroimaging on ECMO. ABI: acute brain injury; EEG: electroencephalography; rSO_2_: regional oxygen saturation; SSEP: somatosensory evoked potential; VA ECMO: venoarterial extracorporeal membrane oxygenation

Standardized neurological monitoring, clinical assessment, and a sedation cessation protocol may increase ABI detection and improve neurological outcomes.^8,12^ In a single-center study (90% VA ECMO), autopsy shortly after ECMO decannulation showed that 68% of ECMO non-survivors had developed ABI.^13^ In another cohort, 9 of 10 brains exhibited ABI at autopsy,^14^ suggesting that ABI incidence is likely higher than clinical detection. Early, accurate ABI detection with standardized neurological monitoring and early interventions is critical for mitigating ABI. Table 3 summarizes current neurological monitoring tools and their evidence (Supplemental Fig. 1), and Table 4 provides the consensus recommendations on neurological monitoring (Fig. 1). A concise review of sedation, disorders of consciousness and seizure is separately summarized in Supplemental File 1.

**Table 4.** Consensus recommendations based on modified Delphi on neuromonitoring/neuroimaging tools during ECMO

Recommendations	References
1. Neuromonitoring/Neuroimaging*	
1.1 Standardized neuromonitoring and neurological expertise for ECMO patients who are at high risk of developing ABI are recommended	^6,32^
1.2. Continuous cerebral oximetry, to follow ongoing trends and early detection of ABI, especially for those with peripheral VA ECMO, who are at risk for differential hypoxia, is recommended	^15,16,18,19^
1.3. Intermittent EEG and SSEP, particularly in comatose patients, are recommended. If available, continuous EEG is especially useful to detect non-convulsive seizures in comatose patients	^25^
1.4. Pupil assessment is recommended. If available, the use of pupillometry should be considered	^7,37^
1.5. Early neuroimaging for ECMO patients at risk of ABI based on physical examination and neuromonitoring tools is recommended	^32,34–36^

ABI: acute brain injury; ECMO: extracorporeal membrane oxygenation; EEG: electroencephalography; SSEP: somatosensory evoked potential; VA: venoarterial.

* Results of the Delphi survey results are available in the Supplementary Material.

## Bedside Management

### Arterial oxygen

The brain depends on aerobic glucose metabolism for energy, with an average cerebral consumption of 3.5 mL oxygen per 100 g of brain tissue per minute. Hyperoxemia (partial pressure of oxygen (PaO_2_) > 100 or 120 mmHg: mild; > 300 mmHg: severe) and hypoxemia (PaO_2_ < 60 or 70 mmHg) are associated with increased mortality in ICU patients, including subjects on ECMO.^41,42^

#### VV ECMO

Limited data exist on early (first 24 h) oxygen targets and neurological outcomes after VV ECMO cannulation. In a single-center observational cohort study, PaO_2_ < 70 mmHg (hypoxemia) was associated with ABI, especially ICH.^43^ There are no data on hyperoxemia as it is not often an issue clinically in VV ECMO patients.

#### VA ECMO

In VA ECMO, when the heart recovers before lung recovery, cerebral hypoxemia (especially of the right side of the brain) may occur due to the “differential oxygenation” (also called “Harlequin Syndrome” or “North–South Syndrome”), which is monitored by arterial blood gases from right radial arterial line, especially for those supported with peripheral VA ECMO. Monitoring of cerebral oxygenation using NIRS may be useful in diagnosing differential oxygenation.^15^

**Figure 2.**
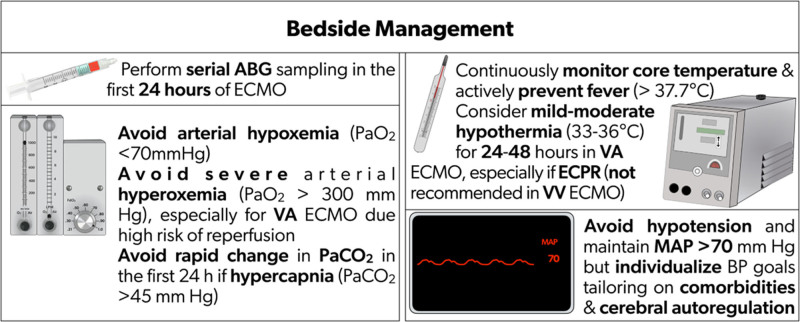
Recommendations for bedside management on ECMO. ABG: arterial blood gas; BP: blood pressure; ECMO: extracorporeal membrane oxygenation; MAP: mean arterial pressure; PaCO_2_: partial pressure of carbon dioxide; PaO_2_: partial pressure of oxygen; VA: venoarterial; VV: venovenous

Severe hyperoxemia (PaO_2_ > 300 mmHg) within 24 h after the cannulation may be associated with ABI and poor neurologic outcomes.^4,42,44^ As optimal oxygenation targets are unknown, it is reasonable to avoid early (within 24 h) severe hyperoxemia and hypoxemia by manipulating the fraction of delivered oxygen from the ECMO sweep gas (Fig. 2). Given the high-quality data are limited, it is crucial to prospectively study the impact of hyperoxemia on ABI and neurological outcomes in VA ECMO as a multi-institutional study with protocolized neurological monitoring and diagnostic ABI adjudication. Importantly, further research is necessary to investigate the impact of hyperoxemia on each major VA ECMO cohort: postcardiotomy shock, ECPR, and post-acute myocardial infarction (AMI) as well as non-AMI cardiogenic shock.

### Arterial carbon dioxide

Severe acidosis and hypercapnia are common before ECMO cannulation, and both are rapidly corrected upon ECMO initiation by adjusting sweep gas flow across the oxygenator. Carbon dioxide is a potent cerebral vasodilator that increases cerebral blood flow^45^ and neuronal metabolic demand.^46^ Prolonged hypercapnia, common in pre-ECMO patients, may impair cerebral autoregulation, leading to high cerebral blood flow and a narrow regulatory pressure window.^40,47^ While high partial pressure of carbon dioxide (PaCO_2_) should be avoided, rapid correction of sustained high PaCO_2,_ particularly soon after ECMO initiation, sometimes leads to rapid hypocapnia; it may cause cerebral vasoconstriction and a decrease in cerebral oxygen delivery, resulting in cerebral ischemia.^46^ Routine use of full-dose anticoagulation therapy at ECMO initiation and thereafter may cause hemorrhagic conversion of an ischemic injury.

#### VV ECMO

In an ELSO registry analysis, a rapid early decrease in PaCO_2_ was independently associated with an increased risk of ICH in ARDS patients with VV ECMO.^48^ An ELSO retrospective study of 11,972 VV ECMO patients showed that those with ΔPaCO_2_ > 50% in the peri-cannulation period were more likely to experience ABI (infarct and ICH).^49^

#### VA ECMO

A higher ΔPaCO_2_ in VA ECMO was associated with ICH in a single-center observational study.^50^ However, an ELSO retrospective study of 3125 ECPR patients showed ΔPaCO_2_ higher in ABI than non-ABI, but ΔPaCO_2_ was not significantly associated with ABI.^4^ These findings are limited by (a) a lack of sensitive, reliable, and readily available diagnostic markers of ABI, (b) retrospective observations, and (c) inconsistent arterial blood gas sampling. Further research with standardized neurological diagnostic/monitoring tools and granular arterial blood gas data is necessary. However, avoiding a large ΔPaCO_2_ > 50% in the peri-cannulation period for both VA and VV ECMO is reasonable.

### Temperature

Inducing hypothermia during ischemia prolongs the tolerance of organs to ischemia, improving neurological outcomes.^51^ Thus, it could be reasonable to use hypothermia in VA ECMO patients where cerebral ischemic and hypoperfusion time is prolonged. This rationale is even more important in patients who have already suffered severe hypoxic-ischemic brain injury, as in ECPR. However, as demonstrated by a meta-analysis of 2643 ECPR patients (35 studies), data on this topic are severely heterogeneous and limited to low-quality evidence.^52^ One randomized controlled trial on cardiogenic shock patients requiring VA ECMO compared moderate hypothermia (33–34 °C) versus normothermia (36–37 °C), showing no mortality difference at 30 days.^53^ This study was limited by (1) insufficient sample size due to inaccurate effect size estimation based on non-ECMO studies, (2) lack of formal neurological assessment, and (3) primary outcome being mortality outcome at 30 days instead of neurological outcomes at 90 or 180 days. The basic and preclinical science on hypothermia in ischemia is strong, and VA ECMO patients have a high incidence of ABI and prolonged absent/low cerebral perfusion. Also, bleeding complications and coagulopathy were similar between those with hypothermia vs. without in a meta-analysis of ECPR patients.^52^ A robust multicenter prospective observation cohort study is needed to test the effect of hypothermia strategically in each major VA ECMO cohort. There is no data on hypothermia in VV ECMO patients.

### Blood pressure

No data exists on early and optimal blood pressure (BP) goals and ABI prevention, especially for stroke or hypoxic-ischemic brain injury, as the timing of ABI is not well-defined during the peri-cannulation period. After acute ischemic stroke, permissive hypertension (BP ≤ 220/120 mmHg) is recommended by the AHA;^54^ it is reasonable to target mean arterial blood pressure (MAP) that can provide adequate cerebral perfusion in the setting of acute ischemic stroke.

Higher BPs lead to increased afterload, which may hinder myocardial recovery (VA ECMO only), particularly when the left ventricle is not vented. In the absence of high-quality data, allowing patients with acute ischemic stroke to autoregulate is reasonable if the heart can tolerate it. After ICH, lower BP (systolic BP < 140 mmHg and MAP < 90 mmHg) is preferred due to anticoagulation-associated ICH.^55^ Cerebral autoregulation function in the setting of non-pulsatile blood flow and ABI is an active research area, and autoregulatory dysfunction may contribute to ABI in ECMO (Supplemental File 2).^56^

Low pulse pressure (< 20 mmHg) in the first 24 h of VA ECMO was associated with ABI.^57^ However, data are weak regarding improving pulse pressure with inotropes, or left ventricle venting in ECMO.^58^ Evidence on BP goals for optimal cerebral perfusion in ECMO patients is sparse. Yet, individualized BP management tailored to dynamic cerebral autoregulation function is likely needed in this complex population. However, evidence as well as related therapeutic actions in this regard are still limited and represent mandatory objectives for future research to enhance ECMO patient management and most likely ABI complications prevention and/or reduction. A summary of consensus recommendations and evidence appears in Table 5 and Supplemental Table 4.

**Table 5.** Consensus recommendations based on modified Delphi on bedside physiological targets for ECMO patients for ABI

Recommendations	References
2. Bedside management*	
2.1. Serial arterial blood gas sampling in the first 24 h of ECMO support is recommended	^4,41,42^
2.2. Avoiding arterial hypoxemia (PaO_2_ < 70 mmHg) is recommended	^4,41,42^
2.3. Avoiding severe arterial hyperoxia (PaO_2_ > 300 mmHg), especially for VA ECMO where reperfusion injury risk is high, is recommended	^42,44^
2.4. For patients with hypercapnia (PaCO_2_ > 45 mmHg), avoiding rapid change in PaCO_2_ within the first 24 h of ECMO support is recommended	^48^
2.5. Continuous monitoring of core temperature and active prevention of fever (> 37.7 °C) are recommended	^51,52^
2.6. Mild-moderate hypothermia (33–36 °C) for 24–48 h in VA ECMO, especially ECPR, is reasonable and may be considered	^51–53^
2.7. Hypothermia in VV ECMO is not recommended	^51–53^
2.8. As optimal ECMO flow and blood pressures are unknown, avoiding hypotension and maintaining mean arterial pressure > 70 mmHg should be considered. Individualized BP goals, based on the patient’s comorbidities, are recommended until further data are available	^55,56^
2.9. Individualized blood pressure management in ECMO patients, tailored to dynamic cerebral autoregulation function may be reasonable	^58^

ECMO: extracorporeal membrane oxygenation; ABI: acute brain injury; ECPR: extracorporeal pulmonary resuscitation; PaCO_2_: partial pressure of carbon dioxide; PaO_2_: partial pressure of oxygen; VA: venoarterial; VV: venovenous.

* Results of the Delphi survey results are available in the Supplementary Material.

## Interventional Neurology, Neurosurgery, and Neurocritical Care

ABI diagnosis in ECMO patients is based on comprehensive neurological assessment and brain imaging. Neurological assessment for acute stroke should include the Glasgow Coma Scale and the National Institutes of Health Stroke Scale. Non-contrast head CT is imperative to rule out ICH with acute neurological exam change. CT angiogram is needed to assess for large vessel occlusion.

### Brain perfusion optimization

Managing intracranial pressure (ICP) and BP contributes to adequate brain perfusion in ABI patients. Elevating the head of the bed by 30 degrees might benefit patients with ABI and elevated ICP.^61^ However, brain oxygenation and circulation improve in the supine position, benefiting perfusion-dependent patients with acute ischemic strokes. The head of the bed could be guided by monitoring surrogate markers of cerebral hemodynamics (i.e., transcranial Doppler ultrasound: cerebral blood flow velocity) and oxygenation (i.e., NIRS: regional saturation).^62,63^ If the heart can tolerate a higher BP, it’s reasonable to target a higher BP target (although individualized BP goal is recommended) to achieve adequate cerebral perfusion pressure, such as permissive hypertension for ischemic stroke. However, increased BP is associated with hematoma extension in ICH, so reducing BP (systolic BP < 140 mmHg) is reasonable, as ECMO patients are usually on full anticoagulation at the time of ICH.

### Managing ischemic stroke

#### Tissue plasminogen activator (tPA)

**Figure 3.**
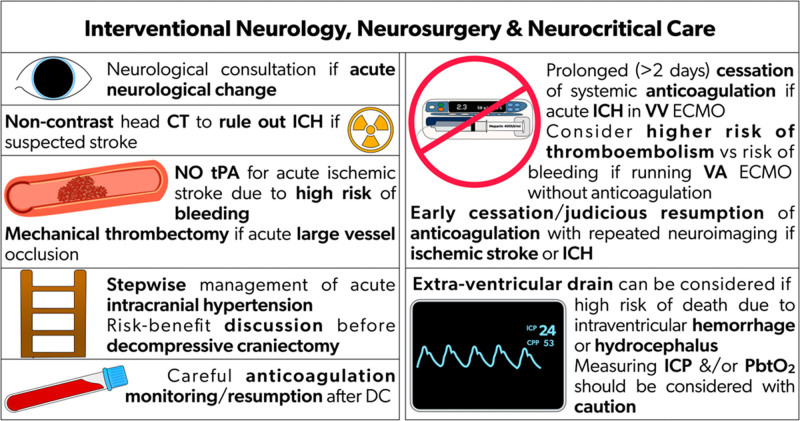
Recommendations for interventional neurology, neurosurgery & neurocritical care on ECMO. CT: computed tomography; ECMO: extracorporeal membrane oxygenation; ICH: intracranial hemorrhage; ICP: intracranial pressure; PbtO_2_: brain tissue oxygenation; tPA: tissue plasminogen activator; VV: venovenous; VA: venoarterial

Non-contrast head CT is imperative to rule out bleeding in acute neurological change, particularly during ECMO. tPA is a time-dependent intervention in acute ischemic stroke. Intravenous tPA in the setting of ECMO carries a high risk of bleeding, especially with systemic anticoagulation and platelet dysfunction. Given these risks, the use of tPA is generally not indicated in ECMO patients. Although there is limited literature specifically addressing this issue, the consensus among experts is to avoid tPA (Fig. 3).

#### Mechanical thrombectomy

CT angiogram is needed to rule out large vessel occlusion, typically accompanied by a CT perfusion scan to assess salvageable penumbra. Mechanical thrombectomy should be pursued for patients with large vessel occlusion detected by CT angiogram (accompanied by a CT perfusion scan to assess salvageable penumbra), by consulting stroke specialists, as tPA is generally not recommended in ECMO.^64^

#### Decompressive craniectomy

Decompressive craniectomy may be indicated in patients with space-occupying lesions with acute intracranial hypertension, such as hemispheric infarction with malignant edema. Hyperosmolar therapy is indicated for cerebral edema.^1^ Systemic anticoagulation monitoring and resumption are necessary post-operatively. Successful craniectomy has been reported for patients on ECMO.^65^ As evidence is limited, the risks versus benefits of such an intervention should be judiciously discussed in a multidisciplinary manner.

### Managing ICH

There are two primary considerations in ICH management. First, preventing hematoma expansion by BP control and discontinuing systemic anticoagulation is recommended. The duration of systemic anticoagulation varies based on the mode of ECMO. VV ECMO may allow anticoagulation discontinuation until decannulation based on multiple reports of heparin-free VV ECMO with a heparin-coated circuit.^66^ In contrast, holding systemic anticoagulation carries a higher risk of thromboembolism with VA ECMO, especially the ECMO circuit.^67,68^ Early cessation without reversal and judicious resumption of anticoagulation with repeated neuroimaging appeared feasible in the cohort of patients with ECMO-associated ischemic stroke and ICH.^37^ Second, surgical or minimally invasive surgery hematoma evacuation may be considered. There is limited data on neurosurgical interventions in ECMO^69^ for patients with no other management options. Neurosurgery may be considered and utilized. Multidisciplinary discussion should be undertaken, involving neurosurgeons and neurologists in decision-making.

#### Intracranial pressure monitoring

While external ventricular drainage may be indicated in patients with ICH with intraventricular extension and hydrocephalus, ECMO is associated with coagulopathy and requires systemic anticoagulation. Therefore, external ventricular drain insertion is a high-risk procedure associated with intra- and post-procedural bleeding.^69^ External ventricular drain may be considered in selected patients at risk of imminent death from intraventricular hemorrhage and hydrocephalus. Monitoring ICP or invasive brain tissue oxygenation may be used in patients at high risk of ICP. Invasive ICP and brain tissue oxygenation have not been shown to improve long-term outcomes and may increase the risk of parenchymal hemorrhage in ECMO patients.

### Cerebral venous sinus thrombosis (CVST)

Diagnosis of CVST requires a high index of suspicion in patients with risk factors for thrombosis, including internal jugular vein cannulation. Particularly, large dual-lumen VV ECMO cannulas may be associated with ABI, possibly due to venous hypertension and cannula-related thrombosis.^70^ Clinical diagnosis is challenging because of varying neurological manifestations, including non-specific symptoms such as headache, seizure, or encephalopathy.^71^ The diagnosis is made with brain CT in ECMO. Systemic anticoagulation is the primary treatment; however, in deteriorating patients, endovascular mechanical thrombectomy in advanced centers may be considered.^72^ Lumbar puncture or other spinal fluid drainage and acetazolamide may be considered for patients with increased ICP, along with anti-edema interventions (raising the head of the bed, hyperosmolar therapy, sedation/analgesia, etc.).^73^ In severe CVST cases with hemispheric cerebral edema, decompressive craniectomy may be considered. A summary of consensus recommendations and evidence is provided in Table 6 and Supplemental Table 5.

**Table 6.** Consensus recommendations based on modified Delphi on neurological intervention and management for ECMO patients with ABI

Recommendations	References
3. Interventional neurology, neurosurgery, and neurocritical care*	
3.1. Neurological consultation for acute neurological change is recommended	^6,12^
3.2. Non-contrast head CT, to rule out ICH in patients with suspected stroke during ECMO, is recommended	^32–36^
3.3. tPA is not recommended for acute ischemic stroke in ECMO, since tPA carries a high risk of bleeding during systemic anticoagulation and platelet dysfunction	
3.4. Mechanical thrombectomy, in cases of acute large vessel occlusion, is recommended	^64^
3.5. Stepwise acute intracranial hypertension management is recommended	^1,61^
3.6. The decision of decompressive craniectomy for stroke, based on a risk–benefit discussion between the multidisciplinary medical team and the patient surrogate, is recommended	^65,69^
3.7. Careful systemic anticoagulation monitoring and resumption after decompressive craniectomy are recommended	^37^
3.8. For acute intracranial hemorrhage during VV ECMO, prolonged (> 2 days) cessation of systemic anticoagulation is recommended	^37,66^
3.9. VA ECMO can be maintained without anticoagulation albeit at a higher risk of thromboembolism. It is recommended that clinicians should balance the risk of anticoagulation and bleeding against the risk of running VA ECMO with no systemic anticoagulation. VV ECMO can be maintained without anticoagulation for a longer period than VA ECMO, given the lower risk of thromboembolism	^67^
3.10. Early cessation and judicious resumption of anticoagulation with repeated neuroimaging with ECMO-associated ischemic stroke and intracranial hemorrhage is recommended	^37,66^
3.11. Data on anticoagulation reversal in ECMO are limited, and therefore no recommendation is provided	
3.12. Extra-ventricular drain placement, in patients with limited management options and high risk of death due to intraventricular hemorrhage and hydrocephalus, may be considered	^69^
3.13. Monitors measuring intracranial pressure and/or brain tissue oxygenation should be considered cautiously, as no data currently suggests that such monitoring improves outcomes in patients with ECMO	^69^

CT: computed tomography; ECMO: extracorporeal membrane oxygenation; ICH: intracranial hemorrhage; tPA: tissue plasminogen activator; VA: venoarterial; VV: venovenous.

* Results of the Delphi survey results are available in the Supplementary Material.

## Neurological Prognostication

### ECPR

Neurological prognostication is imperative in patients supported by ECPR, in which severe hypoxic-ischemic brain injury may occur as a consequence of refractory cardiac arrest and/or due to inadequate ECMO flow and differential hypoxia. It provides families and caregivers critical information and guides treatment decisions based on the likelihood of a meaningful neurological recovery. As the data on neurological prognostication is limited,^74^ a comprehensive approach to prognostication is needed.

Clinical examination plays a pivotal role in prognostication. Practitioners should first rule out potential confounding factors, such as sedatives, significant electrolyte disturbances, and hypothermia, to prevent an overly pessimistic prognosis. Daily clinical/neurological assessments are recommended for patients undergoing targeted temperature management, with the most crucial evaluation conducted after rewarming.^74^ Attention should be given to pupillary and corneal reflexes.^75,76^ Clinicians must exercise caution to mitigate the “self-fulfilling prophecy” bias, which occurs when prognostic test results indicating poor outcomes influence treatment decisions.^77^

A comprehensive prognostication strategy should include electrophysiological tests, the evaluation of biomarkers of ABI, and neuroimaging (Table 7). Notwithstanding, new modalities are under investigation and will hopefully provide additional clues in such a setting regarding early and enhanced detection of ABI as well as prognostication in ECMO patients.^78,79^ An unfavorable neurological outcome in patients without ECMO and cardiac arrest is strongly suggested by at least two indicators of severe ABI. These include the absence of pupillary and corneal reflexes at ≥ 72 h, bilateral lack of N20 cortical waves in somatosensory evoked potentials (SSEP) at ≥ 24 h, highly malignant EEG patterns at > 24 h, neuron-specific enolase levels exceeding 60 μg/L at 48 h or 72 h, status myoclonus ≤ 72 h, and extensive diffuse anoxic injury observed on brain CT/MRI.^74,80^ This approach has not been validated in ECMO patients and has limited evidence.^30^

**Table 7.** Consensus recommendations on neurological prognostication for ECMO patients

Recommendations	References
4. Neurological prognostication*	
4.1. Neurological prognostication for ECPR based on a multimodality, multidisciplinary approach of clinical/neurological examination, electrophysiological tests, and neuroimaging is recommended	^30^
4.2. It is not recommended to use any single factor/tool (e.g. brain imaging only) as the sole indicator for patient prognosis	^30^
4.3. Determination of brain death, based on the presence of devastating brain injury on imaging, neurological examination, and apnea test after considering official recommendations, guidelines, and laws of the specific country and excluding confounding factors is recommended. When an apnea test is challenging, cerebral angiogram or nuclear scan (radionuclide brain scan) are preferred ancillary tests	^75,76,81^
4.4. Frequent meetings and goals of care discussions with the patient surrogate that reflect the patient’s preferences is recommended	^82–84^

ECPR: extracorporeal cardiopulmonary resuscitation.

* Results of the Delphi survey results are available in the Supplementary Material.

Neuron-specific enolase values are often higher in ECMO patients due to ongoing hemolysis.^30,85^ The most accurate neuron-specific enolase threshold for predicting an unfavorable neurological outcome in ECPR remains unknown, possibly exceeding 100 μg/L. There are sparse data on ECMO patients regarding other biomarkers, such as neurofilament light chain or tau. A combination of clinical, biomarker, electrophysiological, and neuroimaging assessment may effectively predict a neurological outcome within the first week following cardiac arrest.^81^ However, limited data exist for this approach in ECMO patients; further research is needed to validate its utility. A summary of consensus recommendations and evidence is provided in Table 7.

### Other neurological diseases

Neurological prognostication in other ABI (non-hypoxic-ischemic brain injury) with ECMO is challenging and relies on less robust data than cardiac arrest. In the context of stroke (ischemic and hemorrhagic), clinical factors impacting outcomes include neurological exam, age, functionality (i.e., modified Rankin Scale), size, and stroke location. For example, age and the location of intracerebral hemorrhage may contribute to neurological prognosis.^86^ However, decisions regarding withdrawal of life-sustaining therapy should be highly individualized with multidisciplinary discussions and considered patient preferences, as data on ECMO patients are sparse.

ICH while the patient is anticoagulated during ECMO carries extremely high mortality and morbidity, as shown in large ELSO registry-based investigations.^87,88^ However, these studies did not account for withdrawing life-sustaining therapy in ECMO. Without data, no recommendations for neurological prognostication in ECMO patients can be made.

### Brain death on ECMO

A systematic review reported that an apnea test could be included in brain-death criteria in ECMO patients by reducing sweep gas flow or adding exogenous carbon dioxide.^89^ When an apnea test is challenging due to hemodynamic/cardiopulmonary instability, a cerebral angiogram or nuclear scan (radionuclide brain scan) is preferred.^89^ We provide recommendations on apnea tests in ECMO patients (Supplemental Fig. 2).

### Goals of care discussion

Goals of care and end-of-life discussions are often culturally influenced or determined. Therefore, it is difficult to propose international guidelines for such. No patient-level research guides communicating with families or managing ECMO discontinuation.^82^ Families of ECMO patients experience significant anxiety, depression, and post-traumatic stress disorder long after hospital discharge.^83^ Frequent family conversations/meetings should focus on informed consent, early goal-setting with timelines and re-evaluation, clear communication, and emotional support with compassion.^82^ Ethics should be discussed openly, including whether to continue or discontinue care and resource allocation issues.^82^ Routine use of ethics consultation within 72 h of cannulation, if the resource is available, can mitigate ethical conflicts by setting clear expectations.^84^ Withdrawal from ECMO should be a structured process involving preparatory family meetings and clinical aspects, including symptom management, technical circuit management, and bereavement support, containing family and staff support.^90^

## Long-Term Outcome and Quality of Life

Sparse information exists on long-term outcomes. Long-term MRI found cerebral infarction or hemorrhage in 37–52% of adult ECMO survivors.^59,60^ Cognitive impairment and neuroradiologic findings were associated.^59,60^ ECMO patients often suffer long-term psychiatric disorders, including organic mental disorders, obsessive–compulsive disorders, and post-traumatic stress disorders.^91^ The incidence of neuroradiologic findings was significantly higher in VA ECMO patients than VV ECMO patients.^59^ Given the high frequency, a routine, long-term, structured, standardized follow-up program is recommended for all ECMO centers. Such programs should encompass disease-specific care for underlying and acquired conditions, focusing on neurological and psychiatric disorders. Program design depends on the availability of institutional and international resources. ECMO centers should adapt follow-up programs their specific patient populations and resources while adhering to the recommendations outlined in Table 8.

**Table 8.** Consensus recommendations on long-term neurological outcomes and follow-ups for ECMO patients

Recommendations	References
5. Long-term outcomes and quality of life*	
5.1. Pre-discharge care	
5.1.1 Clinical examination and use of the modified Rankin Scale before the discharge are recommended. Neuroimaging (preferably conventional MRI brain after decannulation) for those with neurological or cognitive dysfunction is reasonable	^59,60^
5.1.2 Outpatient care planning, with careful consideration of the timing of visits (preferably at 3, 6, and 12 months) after discharge, location of visits (preferably at ECMO clinics or neurologist), and ECMO-related comorbidities and complications (vascular, myopathy, chronic infection, cardiopulmonary recovery) is recommended	^59,60^
5.1.3 Comprehensive education and psychosocial support for patients, family members, and caretakers are recommended	^59,60,92^
5.1.4 Assessment and formulation of a nutritional plan for optimal recovery is recommended	
5.2. Post-discharge care	
5.2.1 Serial neurological assessments and quality of life assessments are recommended	^59,60^
5.2.2 In patients with neurological complications, clinical examination by a neurological specialist, neuroimaging (preferably MRI), and other tailored examinations/tests are recommended	^59,60^
5.2.3 Follow up with disease-specific specialists that are tailored to the underlying disease and comorbidities, including pulmonologist, cardiologist, neurologist, nephrologist, gastroenterologist, and hematologist, is recommended as needed	^59^
5.2.4 Follow up with the primary care physician is recommended	^60^
5.2.5 Establishing a centralized and secure data repository to store patient data that can be shared with outpatient healthcare providers is recommended	^93^

ECMO: extracorporeal membrane oxygenation; MRI: magnetic resonance imaging.

* Results of the Delphi survey results are available in the Supplementary Material.

### Neurological outcomes and quality of life

Assessing ECMO survivors’ quality of life is crucial to understanding the overall impact of ECMO. It is preferable to use internationally recognized, validated tests at standardized intervals. Establishing uniform measures of cognitive function in ECMO patients may clarify outcomes in future studies. Therefore, all patients should have their modified Rankin Scale assessed at discharge and during each follow-up. Additional detailed assessments may be performed based on local practices and patient conditions (e.g., Glasgow Outcome Scale Extended, Montreal Cognitive Assessment). A summary of consensus recommendations and evidence is provided in Table 8 and Supplemental Fig. 3.

## Conclusions

The impact of ABI on morbidity and mortality in ECMO patients is high, and early ABI detection and timely intervention may improve outcomes. Therefore, standardized neurological monitoring and neurological expertise are recommended for ECMO patients. These consensus recommendations and scientific statements serve to guide the neurological monitoring and prevention of ABI, and management strategy of ECMO-associated ABI These recommendations strongly benefit from multidisciplinary care, where it is available, to maximize the chances of favorable long-term outcomes and a good quality of life. Further research on predisposing factors, prevention, neuroimaging and management are ongoing or further required in an attempt to reduce or prevent such dreadful adverse events in ECMO patients.

## Author contributions

S.-M.C. prepared the first draft, led the conceptualization and approach, and finalized the guidelines. A.M.Z. and R.L. provided critical revision and contributed in finalizing the guidelines as co-chairs. J.H. and G.C. provided tables and contributed to the first draft and revisions. M. A. created all figures and supplemental figures. M.A., N.B., J.B., J.E.B., D.B., H.J.D, R.D., A.E., P.T.F., H.F., J.F., A.H., J.-S.J., C.L., G.M, G.P., A.P., J.P., L.R., K.R., D.R.M., D.R., L.S.R., F.S.T., and G.W. were divided into 6 writing groups and prepared each section of the guidelines (6 sections).

## Funding

Dr. Cho is supported by NIH (1K23HL157610, 1R21NS135045). Dr. Brodie received research support from and consults for LivaNova.

## Availability of data and materials

No datasets were generated or analysed during the current study.

## Declarations

### Ethical approval and consent to participate

Not applicable as this is a consensus guidelines article.

### Competing interests

Dr. Cho is a consultant for Hyperfine, Inc. and supported by NIH (1K23HL157610 and 1R21NS135045). Dr. Brodie received research support from and consults for LivaNova. He has been on the medical advisory boards for Xenios, Medtronic, Inspira, and Cellenkos. He is the President-elect of the Extracorporeal Life Support Organization (ELSO) and the Chair of the Executive Committee of the International ECMO Network (ECMONet), and he writes for UpToDate. Dr. Daniel is supported by MH CZ (DRO-VFN64165) and receives consulting honoraria from Abiomed and Resuscitec. Dr. Lorusso received research support from Medtronic and LivaNova, is consultant for Medtronic and Livanova, Member of the Medical Advisory Board of Eurosets and Xenios, and receives speaker fee from Abiomed.
